# Assessment of bacteriophage activity against local strains of *Enterococcus* and *Pseudomonas* in Romania

**DOI:** 10.1186/1471-2334-14-S7-O19

**Published:** 2014-10-15

**Authors:** Alina Cristina Neguț, Oana Săndulescu, Anca Streinu-Cercel, Zemphira Alavidze, Ioana Berciu, Veronica Ilie, Magdalena Lorena Andrei, Dana Mărculescu, Mircea Ioan Popa, Adrian Streinu-Cercel

**Affiliations:** 1 Carol Davila University of Medicine and Pharmacy, Bucharest, Romania; 2 National Institute for Infectious Diseases "Prof. Dr. Matei Balş", Bucharest, Romania; 3 Phage Therapy Center Tbilisi, Georgia

## Background

*Enterococcus faecium* and *Pseudomonas aeruginosa* are part of the ESCAPE pathogens, which have the ability to “escape” the currently available therapeutic options. For these germs alternatives are needed, as is the case of bacteriophage treatment.

## Methods

In this study we used a bacteriophage testing kit containing 4 types of Georgian products: PYO, INTESTI (Eliava BioPreparations, Tbilisi) and PHAGYO, PHAGESTI (JSC "Biochimpharm", Tbilisi) to test the strains of *Pseudomonas* spp. and *Enterococcus* spp. isolated and stored from patients treated in the Adults II ward of the National Institute for Infectious Diseases “Prof. Dr. Matei Balş”, Romania during April 2013 – July 2014.

## Results

We identified 9 strains of *Enterococcus* (7 *E. faecalis*, 1 *E. avium* and 1 *E. faecium*) and 9 strains of *Pseudomonas aeruginosa*. The strains had been isolated mostly from cutaneous wounds (3/9) for *Enterococcus* spp. and from urine (5/9) for *P. aeruginosa*. For *Enterococcus* spp. the rate of susceptibility to PYO phages was 33.33% (3/9), to INTESTI 55.56% (5/9), to PHAGYO 44.44% (4/9) and to PHAGESTI 0% (0/9). We tested the *Enterococcus* ATTC, and it displayed susceptibility to PYO, INTESTI and PHAGYO.

For *Pseudomonas* spp. the rate of susceptibility to PYO phages was of 66.67% (6/9), INTESTI 88.89% (8/9), PHAGYO 55.56% (5/9), PHAGESTI 44.44% (4/9). We tested the *Pseudomonas* ATTC, and it displayed susceptibility to all the bacteriophage products tested.

Performing the ANOVA test for *Enterococcus* spp. we identified a statistically significant correlation between susceptibility to ampicillin vs. PYO phages (p=0.034) and vs. INTESTI phages (p=0.024). For *P. aeruginosa* a correlation was identified between susceptibility to ceftazidime and PYO phages (p=0.029).

## Conclusion

Despite the fact that PYO and PHAGYO do not contain phages for *Enterococcus* spp., their activity against it was similar to that of INTESTI. PYO and PHAGYO showed activity on *Enterococcus* ATCC, too. For *P. aeruginosa* the rate of susceptibility was very high for phages, and quite low for almost all antibiotics tested. We intend to develop further studies for testing a higher number of strains and for assessing a potential synergy for co-administration of antibiotics and bacteriophages.

